# A Peculiarity in the Human Hybrid

**Published:** 1880-10

**Authors:** Harvey L. Byrd

**Affiliations:** 225 Gilmor St., (Harlem Square,) Baltimore, Md.


ARTICLE II.

### A PECULIARITY IN THE HUMAN HYBRID.

BY HARVEY L. BYRD, A. M., M. D., ETC., ETC., BALTIMORE, MD.

A curious and interesting fact in regard to the admixture of blood as it occurs in the human hybrid—mulatto, seems, so far as I have learned, to have escaped the notice of those who have written on the subject of the interbreeding of the caucasian and negro races; and it is therefore, deemed of sufficient moment to occupy a few brief paragraphs in the journal at this time.

Every thing that is capable of throwing any additional light upon the great obscurity which has so deeply shrouded the origin and history of the human races during the eons of the existence of some of them, at least, should, I think, be considered of sufficient value and importance to be placed upon permanent record for the benefit of such as are, and may be engaged in the study and investigation of the intensely interesting subject of Ethnography! As stated in a previous article in this journal, page 375, my opportunities for studying the phenomena developed in the human hybrid, have been confined exclusively to the offspring of the white man and negro woman, but these have developed many curious and interesting facts both in their mental and physical characteristics, well worthy of extended study and investigation by those who would more fully learn the physiological laws governing hybridity in the human races. Anatomists are aware of the great and radical differences and distinctions which so widely separate and indellibly mark the white and black races; and are in this country, at least, familiar with the structure of the mulatto also; but a knowledge of all this is not sufficient to account for certain physiological facts which seem unique, and to belong to the human hybrid peculiarly and alone; but I must proceed to speak of that one now, which more immediately evoked attention to this article. Extended observation and careful notation in the direction already indicated, show that from *sixty-five* to *seventy per cent.* of the children begotten of negro women, by white men, are *female!* Whereas, the average births in the late slave-holding states of this country, where my observations were chiefly made, in the unmixed caucasian and negro races, were from one hundred and four, to one hundred and six males, to one hundred females, in the former; and from one hundred and one to one hundred and three males to one hundred females, in the latter. In some few localities the births seemed to have been about *equal*, as between male and female, in the negro race. The percentage of males in this race, in all cases, where my statistics were obtained, was very small as already shown by the numbers above. That there *are* laws governing every function in the animal organism, is too well known to enlightened scientists to require any argument to establish their existence and their operation. In fact, it would be about as reasonable to doubt one's own existence at the present day, as to question the universality and unvarying perpetuity of such law or laws! In our present limited acquaintance with the secret operations of nature, we can furnish no good reason for the large per centage of the female sex in the human hybrid; but its significance should not be overlooked nor disregarded on that account; if we would hope to be able to read in future, its meaning for good!

Such observations and comparisons as I have been able to make in regard to the *Mule*, have led me to believe also that a *large percentage of that hybrid is female!* I have thought from time to time, I would interrogate some gentlemen of experience in breeding mules, as to whether the percentage does not rise quite as high in them as in the mulatto? Should this paragraph meet the eye of any one of experience and observation on this point, such information, confirmatory or not, of my limited observation, as they might think proper to impart, would be thankfully received and duly appreciated. Could not some of our confreres in the *Southwest* furnish the information we desire to have on this point? At a future time, I may take occasion to add something on the tendency of the human hybrid to certain diseases, some of which are not inherited from either race; and on the *impossibility* of *its* perpetuation as a *variety* of the genus homo! and thus attempt to show another parallelism between the mulatto and the mule.

225 Gilmor St., (Harlem Square,) Baltimore. September, 1880.

